# On the inverse association between the number of QTL and the trait-specific genomic relationship of a candidate to the training set.

**DOI:** 10.1186/s12711-024-00940-4

**Published:** 2024-12-13

**Authors:** Christian Stricker, Rohan L. Fernando, Albrecht Melchinger, Hans-Juergen Auinger, Chris-Carolin Schoen

**Affiliations:** 1 agn Genetics, Boertjistrasse 8b, Davos, 7260 Switzerland; 2 Department of Animal Science, Iowa State University, Kildee Hall, Ames, 50011 IA USA; 3 Plant Breeding, School of Life Sciences, Technical University of Munich, Liesel-Beckmann-Strasse 2, Freising, 85354 Germany

## Abstract

**Background:**

Accuracy of genomic prediction depends on the heritability of the trait, the size of the training set, the relationship of the candidates to the training set, and the $\text {Min}(N_{\text {QTL}},M_e)$, where $N_{\text {QTL}}$ is the number of QTL and $M_e$ is the number of independently segregating chromosomal segments. Due to LD, the number $Q_e$ of independently segregating QTL (effective QTL) can be lower than $\text {Min}(N_{\text {QTL}},M_e)$. In this paper, we show that $Q_e$ is inversely associated with the trait-specific genomic relationship of a candidate to the training set. This provides an explanation for the inverse association between $Q_e$ and the accuracy of prediction.

**Methods:**

To quantify the genomic relationship of a candidate to all members of the training set, we considered the $k^2$ statistic that has been previously used for this purpose. It quantifies how well the marker covariate vector of a candidate can be represented as a linear combination of the rows of the marker covariate matrix of the training set. In this paper, we used Bayesian regression to make this statistic trait specific and argue that the trait-specific genomic relationship of a candidate to the training set is inversely associated with $Q_e$. Simulation was used to demonstrate the dependence of the trait-specific $k^2$ statistic on $Q_e$, which is related to $N_{\text {QTL}}$.

**Conclusions:**

The posterior distributions of the trait-specific $k^2$ statistic showed that the trait-specific genomic relationship between a candidate and the training set is inversely associated to $Q_e$ and $N_{\text {QTL}}$. Further, we show that trait-specific genomic relationship between a candidate and the training set is directly related to the size of the training set.

**Supplementary Information:**

The online version contains supplementary material available at 10.1186/s12711-024-00940-4.

## Background

Genomic prediction is widely used in plant and animal breeding for genetic improvement of populations by combining genotypic and phenotypic data to obtain more accurate predictions of breeding values at an earlier age than was possible when only phenotypic data and pedigree were used for prediction [1, 2]. Genomic Best Linear Unbiased Prediction (GBLUP) and Bayesian “alphabet” methods are widely used for genomic prediction [3–5]. These methods have been shown to be consistent and, thus, given sufficient data, would yield similar accuracies [6–8]. It is well known that the accuracy of genomic prediction depends on the heritability of the trait, the size of the training set, the relationship of the candidates to the training set, and the number of marker loci used for prediction [9–12]. Early formulas for accuracy, e.g., formula (1) in [11], showed that accuracy of genomic prediction was inversely related to the number of marker loci used for prediction, where the markers were assumed to be independent. This inverse association follows from the fact that genomic prediction is based on estimated effects of the markers, leading to the accumulation of the errors of estimation in the predicted value. However, when large numbers of markers are used for prediction, they cannot be assumed to be independent, because of linkage disequilibrium (LD) between the markers. To account for this, Goddard [10] introduced the concept of the effective number of markers used for prediction ($M_e$). Now, accuracy is inversely related to $M_e$ and not to the actual number of markers as shown, for example, in formula (1) in [12]. When variable selection is used, Daetwyler et al. [12] showed that accuracy of prediction is inversely related to $\text {Min}(N_{\text {QTL}},M_e)$, where $N_{\text {QTL}}$ is the number of QTL. Even when $N_{\text {QTL}} < M_e$, it is possible that, due to LD, not all QTL segregate independently. Thus, we define $Q_e$ to be the number of independently segregating chromosomal segments with at least one QTL, and from hereon, we will refer to $Q_e$, which is $\le N_{\text {QTL}}$, as the effective number of QTL. Suppose the positions of the $Q_e$ independently segregating chromosomal segments with at least one QTL are known and their effects are estimated. Then, as mentioned above, accuracy of prediction will be inversely related to $Q_e$ because of the accumulation of errors from estimation. When the positions of these $Q_e$ independently segregating chromosomal segments are not known, markers are used for prediction. Then, it is possible that the number *s* of trait-specific markers that are necessary to best explain the genetic variability is larger than $Q_e$ due to incomplete LD between the markers and the independently segregating chromosomal segments with QTL. As the marker density increases, the value of *s* is expected to get closer to that of $Q_e$, and thus for simplicity, we will not always distinguish between *s* and $Q_e$.

In this paper, we will show that there is an additional contribution to the inverse association between the accuracy of prediction and $Q_e$. Given genomic data, even candidates that are not related by pedigree, i.e., they do not share alleles identical by descent, can be genomically related through genetic similarity, i.e., they share alleles identical by state. We will show here that the genomic relationship between a candidate and a training set of a given size is also inversely associated with $Q_e$. As genomic relationship is directly related to the accuracy of prediction, an inverse association between $Q_e$ and the genomic relationship will be an additional contribution to the inverse association between accuracy of prediction and $Q_e$.

Several statistics have been proposed to summarize the relationships between a candidate and a set of individuals [8, 13, 14]. For example, the maximum value of the pedigree-based additive relationships between the candidate and the individuals in a training set was used in [13], while both the mean and maximum values of genomic relationships were used in [14]. In this paper we discuss how two of these statistics are inversely associated with $Q_e$. A computer simulation was used to demonstrate this association in a maize-breeding context.

The inverse association mentioned above may seem counter intuitive for two reasons. First, it holds only for genomic relationships and is not true for pedigree-based relationships. Second, it refers to the relationship between an individual and a set of individuals. We will show below that the relationship between a candidate and the individuals in the training set summarized by the maximum value of the genomic relationships is inversely associated with the number of markers used to compute these genomic relationships.

To see why this inverse association holds only for genomic relationships and is not true for pedigree-based relationships, we need to understand how these two types of relationships differ from each other. The pedigree-based additive relationship coefficient between two individuals is twice the probability that randomly sampled homologous genes from the two individuals are identical by descent [15]. Thus, conditional on the pedigree, this coefficient is fixed, i.e, given the pedigree, it is not a random variable. On the other hand, the genomic relationship between two individuals, conditional on the same pedigree relationship with each other, can be thought of as a random variable. Consider the ideal situation where genotypes are available at the QTL. Let $\textbf{z}_i$ and $\textbf{z}_j$ denote vectors of these genotypes for individuals *i* and *j* that have been centered and scaled to have means of zero and variances of one. It can be shown that the cross product of the genotypes from *i* and *j* at any locus *k* is a random quantity that has expected value equal to the pedigree-based additive relationship coefficient: $a_{ij}$ [4, 9]. Thus, the genomic relationship, which can be computed as the mean of these cross products:

1 $$\begin{aligned} g_{ij} = \frac{\sum _{k=1}^q z_{ik}z_{jk}}{N_{\text {QTL}}}, \end{aligned}$$

also has expectation equal to $a_{ij}$. Further, from equation 1, it can be seen that the variance of $g_{ij}$, which is trait-specific, is inversely associated with the number $N_{\text {QTL}}$ of QTL. But, the maximum value of the genomic relationships of an individual with a set of individuals will be proportional to the variance of these relationships, which now we can see has an inverse association to the number of genotypes used to compute the $g_{ij}$. This will not be true for the maximum value of $a_{ij}$, because it is not computed from genotypes and is a fixed quantity given the pedigree. Further, the inverse association with $Q_e$ will also not be true for the mean value of $g_{ij}$, because the mean does not have an inverse relationship to $N_{\text {QTL}}$. In the Methods section of this paper, we will show that the $k^2$ statistic proposed in [8] has an inverse association with $Q_e$. In that section, we will also show mathematically how $k^2$ is related to the accuracy of genomic prediction.

Kizilkaya et al. [16] have shown by computer simulation that genomic prediction under additive inheritance is more accurate for traits that are determined by a smaller number of QTL than for those determined by a larger number. In Table 1 of their paper, where the candidates were in the training set, the accuracy of prediction using 50k markers did not depend on the number of QTL underlying the trait. In their Table 2, however, when candidates were not in the training set, the accuracy using 50k markers did depend on the number of QTL. We will show how the inverse association of $k^2$ with the number of QTL underlying the trait holds only when the candidate is not in the training set. If the candidate is in the training set, the maximum value of $g_{ij}$ will be $g_{ii} = 1.0$ and will, therefore, not depend on the number of QTL. Thus, this explains why, in [16], the accuracy of genomic prediction did not depend on the number of QTL when the candidates were in the training set but did depend on the number of QTL when candidates were not in the training set. In this paper, we will use the simulation results from [16] to disentangle the two factors that contribute to the inverse association between the accuracy of prediction and $Q_e$, where one of these comes from the accumulation of errors of estimation and the other comes from the inverse association of $Q_e$ with the genomic relationship of the candidate to the training set.

## Methods

### $k^2$ Statistic and Predictability

Several statistics have been considered to summarize the genomic relationships between a candidate and the individuals in the training set [8, 13, 14]. Here, we will focus on the $k^2$ statistic proposed by [8] for this purpose. Their statistic is based upon the unique decomposition of the vector $\textbf{k}$ of SNP covariates of the candidate into two vectors, $\textbf{k}_p$ and $\textbf{k}_r$:

$$\begin{aligned} \textbf{k} = \textbf{k}_p + \textbf{k}_r, \end{aligned}$$

where $\textbf{k}_p$ is a linear combination of the SNP covariate vectors of the individuals in the training set, and $\textbf{k}_r = \textbf{k} - \textbf{k}_p$, which can be shown to be orthogonal to the SNP covariate vectors of the individuals in the training set. Analogous to the decomposition of $\textbf{k}$, the genomic breeding value $u = \textbf{k}'\varvec{\alpha }$ can be decomposed as

$$\begin{aligned} u&= \textbf{k}'\varvec{\alpha } \nonumber \\ &= \textbf{k}'_p\varvec{\alpha } + \textbf{k}'_r\varvec{\alpha } \nonumber \\ &= u_p + u_r \nonumber , \end{aligned}$$

where $\varvec{\alpha }$ is the vector of unknown substitution effects of the SNPs. As will be shown later, because $\textbf{k}_r$ is orthogonal to the SNP vectors of the training individuals, the component $u_r$ of *u* cannot be predicted, using the phenotypes of the individuals in the training set, and only the component $u_p$ of *u* can be predicted, using these phenotypes. We define the predictability of *u* to be the squared correlation between *u* and $u_p$, where $u_p$ is the component of *u* that can be predicted using the phenotypes in the training set. Assuming that $\text {Var} (\varvec{\alpha }) = \textbf{I}\sigma ^2_{\alpha }$, the predictability of *u* is:

2 $$\begin{aligned} \text {Cor}^2(u,u_p)&= \frac{\text {Cov}^2(u,u_p)}{\text {Var(u)}\text {Var}(u_p)} \nonumber \\ &= \frac{(\textbf{k}'_p\textbf{k}_p\sigma ^2_{\alpha })^2}{(\textbf{k}'\textbf{k}\sigma ^2_{\alpha })(\textbf{k}'_p\textbf{k}_p\sigma ^2_{\alpha })} \nonumber \\ &= \frac{\textbf{k}_p'\textbf{k}_p}{\textbf{k}'\textbf{k}} \nonumber \\&= k^2, \end{aligned}$$

and it is identical to the statistic proposed in [8]. Here, $\textbf{k}_p'\textbf{k}_p\sigma ^2_{\alpha }$ is the variance of $u_p$ and $\textbf{k}'\textbf{k}\sigma ^2_{\alpha }$ is the variance of *u*, and thus, $k^2$ gives the proportion of the variance of *u* that is due to the component $u_p$, which is the only component of *u* that is correlated to the phenotypes in the training set.

Thus, the ratio $k^2$ quantifies the relationship between the candidate and the training set in three ways: 1) it is the squared correlation between *u*, which is what we want to predict, and $u_p$, which is the only component of *u* that we can predict using the phenotypes in the training set; 2) it is the proportion of the variance of *u* that is due to the component $u_p$; and 3) it quantifies how well the vector $\textbf{k}$ of marker covariates can be expressed as a linear combination of the SNP covariate vectors of the individuals in the training set.

### Inverse association of $k^2$ to the effective number $Q_e$ of QTL

In their paper [8], it was assumed that all *m* available markers are used for prediction, and therefore, their measure was not trait specific. However, we have already recognized that accuracy of prediction is not inversely related to *m* but to the effective number $Q_e$ of QTL, which is trait-specific. Suppose, for some trait, *s* trait-specific markers are available that best explain the variability due to the QTL for this trait. When the number *s* of these trait-specific markers is smaller than the number *n* of training individuals, it is possible that the row rank of the $n\times s$ trait-specific matrix of marker covariates of the training set is also *s*. In that case, for any $\textbf{k}$, the *s* elements that correspond to the *s* trait-specific markers can be written as a linear combination of the $n\times s$ trait-specific marker covariate matrix of the training individuals, and thus the trait-specific $k^2_{s}$ will be 1.0 for any candidate. However, when $s> n$, $k^2_s$ may be smaller than 1.0. Even in this situation, $k^2_s$ can be 1.0, for example when the candidate is in the training set. The number *s* of trait-specific markers is expected to be greater than or equal to $Q_e$, and thus, $k^2_{s}$ is expected to be higher for traits with a smaller value of $Q_e$. It follows that $k^2$, which is computed using all *m* markers, would be less than or equal to $k^2_{s}$ computed from the set of *s* trait-specific markers.

Note that in variable-selection methods, such as BayesC$\pi$ [5], inferences are based on Markov Chain Monte-Carlo (MCMC) samples of all unknowns, including the proportion $(1 - \pi ) = \frac{s}{m}$ of trait-specific markers and the actual set of the *s* trait-specific markers that capture the variablity due to the QTL. Thus, trait-specific values of $k^2_s$ can be calculated without knowing $\pi$ nor the set of *s* trait-specific markers. These samples can be used to draw inferences about $k^2_s$ from its posterior distribution. This is similar to [17], where Bayesian multiple regression models were used to draw inferences about genomic-relationship matrices from MCMC samples. The differences between this paper and [17] are as follows. In this paper, the genomic relationship quantified by $k^2_s$, is between one candidate and an entire set of individuals (e.g. the training set). Thus, as shown here, this genomic relationship is inversely associated with $Q_e$ and directly with *n*, the number of individuals in the set. In [17], however, the genomic relationships were quantified by a trait-specific, genomic-relationship matrix, i.e., they were the traditional genomic relationships between pairs of individuals. The genomic relationship between a pair of individuals is not related to *n*, nor is it expected to be associated with $Q_e$.

We hypothesize that two traits with the same heritability can have different accuracies due to one of them having a smaller value for $Q_e$, resulting in higher values for $k^2_s$. As described later, a computer simulation was used to test this hypothesis.

### Accuracy of BLP and $k^2$

Here we will show in detail how the accuracy of best linear prediction (BLP) is related to $k^2$. The BLP of the genomic breeding value, $u = \textbf{k}'\varvec{\alpha }$, can be written as $\hat{u} = \textbf{k}'\hat{\varvec{\alpha }}$, where $\hat{\varvec{\alpha }}$ is the BLP of $\varvec{\alpha }$. This BLP is obtained by modeling phenotypes, as

3 $$\begin{aligned} \textbf{y}= & \textbf{X}\varvec{\alpha } + \textbf{e}, \end{aligned}$$

where, for simplicity, we have ignored all other non-genetic effects on $\textbf{y}$, $\textbf{X}$ is an $n\times m$ matrix of centered marker covariates, and $\textbf{e}$ is the vector of environmental effects. In most current practical situations, the matrix $\textbf{X}$ has many more columns than rows ($m>n$). Thus, it is customary to treat $\varvec{\alpha }$ as a random vector with null means and covariance matrix $\textbf{I}\sigma ^2_\alpha$. Then, $Var(\textbf{y} | \textbf{X}) = \textbf{V} =\textbf{G}\sigma ^2_\alpha + \textbf{I}\sigma ^2_e$, where $\textbf{G} = \textbf{XX}'$, and BLP of $\varvec{\alpha }$ is

4 $$\begin{aligned} \hat{\varvec{\alpha }}= & \text {Cov}(\varvec{\alpha }, \textbf{y})\textbf{V}^{-1}\textbf{y} \nonumber \\= & \sigma ^2_{\alpha }\textbf{X}'\textbf{V}^{-1}\textbf{y}. \end{aligned}$$

This BLP of $\varvec{\alpha }$ can be used to get the BLP of the breeding value of the candidate with the centered genotype covariate vector $\varvec{k}$ as

$$\begin{aligned} \hat{u}= & \textbf{k}'\hat{\varvec{\alpha }}\\= & \sigma ^2_{\alpha }\textbf{k}'\textbf{X}'\textbf{V}^{-1}\textbf{y}\\= & \sigma ^2_{\alpha }\textbf{c}'\textbf{V}^{-1}\textbf{y}, \end{aligned}$$

where $\textbf{k}'\textbf{X}' = \textbf{c}'$ is a vector with elements that are proportional to the genomic relationships between the candidate and the training set. Using properties of BLP (e.g. [18]), the reliability of this predictor can be written as

$$\begin{aligned} \text {Cor}^2(u,\hat{u})= & \frac{Var(\hat{u})}{Var(u)} \\= & \frac{\sigma ^2_{\alpha }\textbf{c}' \textbf{V}^{-1}\textbf{V}\textbf{V}^{{-1}{'}} \textbf{c}\sigma ^2_{\alpha }}{\textbf{k}' \sigma ^2_{\alpha } \textbf{k}}\\ = & \frac{\sigma ^2_{\alpha }\textbf{c}' \textbf{V}^{-1}\textbf{c}}{\textbf{k}' \textbf{k}}. \end{aligned}$$

It is easy to see that if the candidate is genomically unrelated to the training set, i.e., $\textbf{c}'=\textbf{0}'$, then the reliability of prediction is null. Recall that we previously decomposed *u* as $u = u_p + u_r$ and claimed that $u_r$ cannot be predicted. This result follows from the decomposition of $\textbf{k}$ into $\textbf{k}_p$ and $\textbf{k}_r$ in [8], where $\textbf{k}_p$ was defined as

5 $$\begin{aligned} \mathbf {k_p} = \textbf{X}'(\textbf{X}\textbf{X}')^-\textbf{X}\textbf{k}, \end{aligned}$$

which is the projection of $\textbf{k}$ on to the row space of $\textbf{X}$, and $\textbf{k}_r$ as

$$\begin{aligned} \mathbf {k_r}' = \textbf{k}' - \mathbf {k_p}', \end{aligned}$$

which can be seen to be orthogonal to the rows of $\textbf{X}$. Further, from equation (4) we can see that $\hat{\varvec{\alpha }}$ is in the column space of $\textbf{X}'$, which is the row space of $\textbf{X}$. Thus, the BLP of *u* becomes

6 $$\begin{aligned} \hat{u}&= \textbf{k}'\hat{\varvec{\alpha }} \nonumber \\ &= (\textbf{k}'_p + \textbf{k}'_r) \hat{\varvec{\alpha }} \nonumber \\ &= \textbf{k}'_p \hat{\varvec{\alpha }} + \textbf{k}'_r \hat{\varvec{\alpha }} \nonumber \\ &= \hat{u}_p + \hat{u}_r \nonumber \\&= \hat{u}_p, \end{aligned}$$

because $\textbf{k}'_r$ is orthogonal to the rows of $\textbf{X}$, which leads to $\textbf{k}'_r\hat{\varvec{\alpha }} = 0$. Alternatively, we see below that $u_r$ is uncorrelated with $\textbf{y}$:

7 $$\begin{aligned} \text {Cov}(u_r,\textbf{y})&= \text {Cov}(\textbf{k}'_r\varvec{\alpha },\textbf{X}\varvec{\alpha } + \textbf{e}) \nonumber \\ &= \textbf{k}'_r\text {Var}(\varvec{\alpha })\textbf{X}' \nonumber \\&= \textbf{k}'_r\textbf{X}'\sigma ^2_{\alpha } \nonumber \\&= \textbf{0}', \end{aligned}$$

because $\varvec{\alpha }$ is uncorrelated with the vector $\textbf{e}$ of residuals, and $\textbf{k}'_r$ is orthogonal to the rows of $\textbf{X}$. Thus, $\textbf{y}$ is not useful to predict $u_r$.

What we see from the above result is that although we would like to predict *u*, the information in $\textbf{y}$ is useful only to predict $u_p$. This justifies our definition of predictability as the square of the correlation between *u* and $u_p$. We show below how the predictability, which from equation (2) equals $k^2$, is related to the reliability of $\hat{u}$, which is the square of the correlation between *u* and $\hat{u}$.

Now, because $\hat{u} = \textbf{k}'_p\hat{\varvec{\alpha }} = \hat{u}_p$, the reliability of $\hat{u}$ can be written as

8 $$\begin{aligned} Cor^2(u,\hat{u})= & \frac{Var(\hat{u})}{Var(u)} \nonumber \\= & \frac{Var(\hat{u}_p)}{Var(u)} \nonumber \\= & \frac{\mathbf {k_p}'Var(\hat{\varvec{\alpha }})\mathbf {k_p}}{\textbf{k}'Var(\varvec{\alpha })\textbf{k}} \nonumber \\= & \frac{\mathbf {k_p}'Var(\hat{\varvec{\alpha }})\mathbf {k_p}}{\sigma ^2_{\alpha }\textbf{k}'\textbf{k}}. \end{aligned}$$

Suppose the size of the training set is increased without changing the row rank of $\textbf{X}$. Then, the predictability will not change because the row space of $\textbf{X}$ is the same and the values of $\textbf{k}_p$ and $\textbf{k}_r$ will remain unchanged. But, the reliability of $\hat{u}$ will increase, because the error of estimating $u_p$, the component of *u* that is correlated with the phenotypes, decreases as the number of phenotypes increases. Thus, at some point, the reliability of $\hat{u}$ will reach its upper bound.

To understand what this upper bound is, consider the reliability of $\hat{u}_p$:

9 $$\begin{aligned} Cor^2(u_{p},\hat{u}_{p})= & \frac{\mathbf {k_p}'Var(\hat{\varvec{\alpha }})\mathbf {k_p}}{\mathbf {k_p}'Var(\varvec{\alpha })\mathbf {k_p}} \nonumber \\= & \frac{\mathbf {k_p}'Var(\hat{\varvec{\alpha }})\mathbf {k_p}}{\sigma ^2_{\alpha }\mathbf {k_p}'\mathbf {k_p}}, \end{aligned}$$

the maximum value of which is 1.0. This implies that the upper bound of $\mathbf {k_p}'Var(\hat{\varvec{\alpha }})\mathbf {k_p}$ is $\sigma ^2_{\alpha }\mathbf {k_p}'\mathbf {k_p}$. Thus it follows that the upper bound for the reliability in (8) is:

10 $$\begin{aligned} k^2 = \frac{\mathbf {k_p}'\mathbf {k_p}}{\textbf{k}'\textbf{k}}. \end{aligned}$$

This is identical to the predictability in equation (2), which we defined as the squared correlation between what we want to predict and what we can predict: $\text {Cor}^2(u,u_p)$.

The predictability or upper bound of reliability in (10) can be computed based on a candidate’s marker covariate vector and its projection onto the row space of $\textbf{X}$, the marker covariate matrix used to obtain $\hat{\varvec{\alpha }}$. Note that if the vector of marker covariates $\textbf{k}'$ for our candidate is in the row space of $\textbf{X}$, $\textbf{k}_p$ will be equal to $\textbf{k}$ and $k^2=1$. On the other hand, if $\textbf{k}'$ is not in the row space of $\textbf{X}$, $k^2 < 1.0$.

### Simulation

In a study involving Maize hybrids (Chris Schoen, pers. comm.), the accuracy of prediction obtained with the same training set was different for two traits that had the same heritability in the same set of candidates. An explanation for this could be the inverse association between $Q_e$ and the genomic relationship of a candidate with the individuals in the training set. To determine whether this explanation is valid, marker data from a plant-breeding experiment were used to simulate a mating scheme relevant to plant breeding, as described below.

#### Pedigree structure

**Fig. 1:**
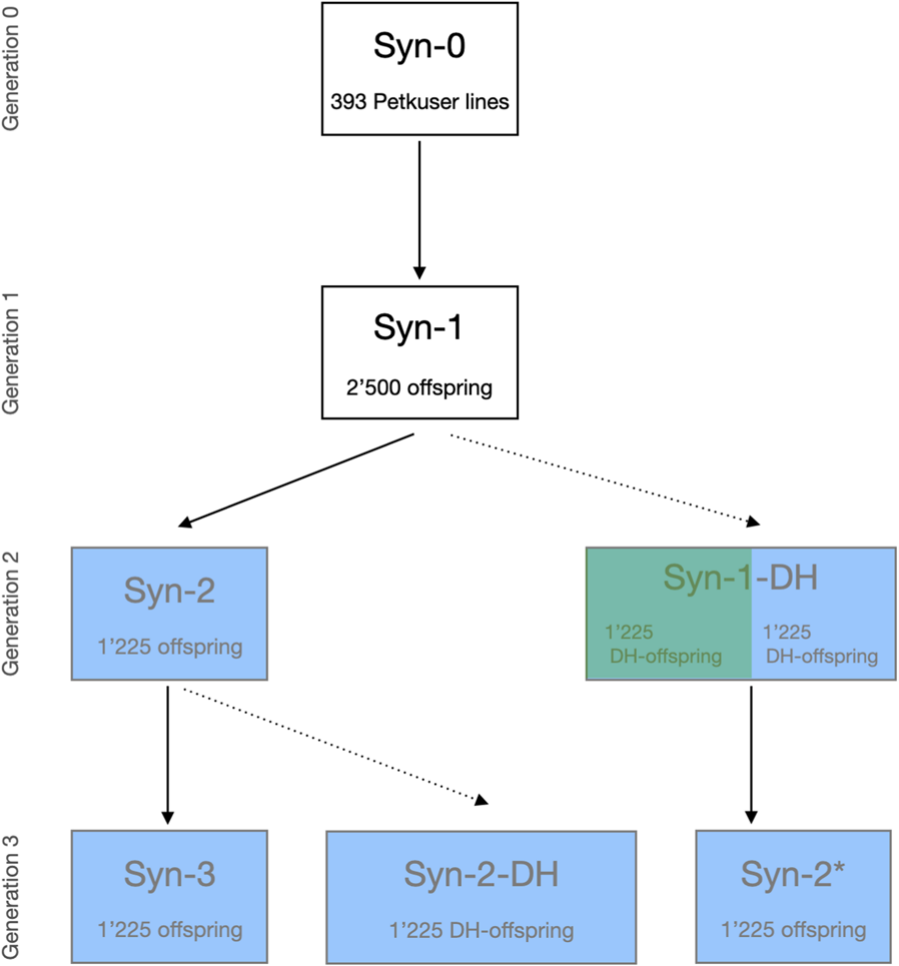
Pedigree structure for the simulated three-generational data. Actual genotypes of 393 Petkuser doubled haploid (DH) maize lines were used as generation 0 (Syn-0). Syn-1 in generation 1, Syn-2 in generation 2 and Syn-3 and Syn-2* in generation 3 were produced by random mating without selfing indicated by solid arrows leading from parental to offspring generations. Syn-1-DH in generation 2 and Syn-2-DH in generation 3 were produced as DH lines originating from a gamete of a randomly selected parent. The simulation of DH lines is indicated by dotted arrows. 250 candidates were randomly sampled from each of the generations coloured in blue. 1,225 DH lines were randomly chosen for training from Syn-1-DH (coloured in green), these lines were used to identify markers with non-null effects

Genotypic and phenotypic values were simulated using the XSim2 software package [19], according to the mating scheme in Fig. 1. 393 doubled haploid lines from the German maize landrace Petkuser as detailed by [20] were used as generation 0 and referred to as Syn-0. Generation Syn-1 was obtained by mating a random sample of 100 DH lines from Syn-0, with 50 lines serving as females and 50 as males in a factorial mating design with one progeny per cross, to produce a total of 2,500 S0 plants. From this set, 70 S0 plants were randomly selected (35 females and 35 males) to generate again one progeny per cross, resulting in a total of 1,225 S0 plants in Syn-2. This procedure was repeated to generate another 1,225 S0 plants in generation Syn-3. The 2,450 DH lines in generation Syn-1-DH and the 1,225 DH lines in generation Syn-2-DH were obtained by doubling one randomly produced gamete from each of the S0 plants in generation Syn-1 and Syn-2, respectively. The lines for the training set (coloured in green in Fig. 1) were randomly selected from Syn-1-DH. In parallel, we sampled 70 DH lines from Syn-1-DH to produce generation Syn-2* using the same procedure as applied for generating generation Syn-2 and Syn-3. Note that the training set was drawn independently of whether an individual was a parent of a candidate in Syn-2*. Only the phenotypes and genotypes of the training lines in Syn-1-DH were used to identify the set of markers with non-null effects.

#### Genotypes and phenotypes

Three scenarios, with 10, 100, and 1,000 QTL underlying a quantitative trait with the same heritability (see below), were simulated. Each scenario was repeated five times. To reduce the computational burden, only the first two maize chromosomes were considered, i.e. 47,265 loci on the first chromosome of length 3.07 Morgans and 35,329 loci on the second chromosome of 2.24 Morgans, resulting in a total of 82,593 loci. Either 10, 100, or 1,000 loci were randomly selected from these 82,593 loci as QTL. Their effects were sampled from a standard normal distribution and were assumed to be additive. Random residual effects were sampled from a normal distribution with variance equal to the genetic variance in the base population, resulting in a heritability of 0.5.

### Inference about $k^2_s$ using BayesC$\pi$

The Bayesian Regression model BayesC$\pi$ [5] was used to sample the trait-specific markers that best captured the variability due to the QTL. The phenotypic values were modeled as

11 $$\begin{aligned} \textbf{y} = \textbf{1}\mu + \textbf{X} \mathbf {\alpha } + \textbf{e}, \end{aligned}$$

where $\textbf{y}$ is the *n* x 1 vector of trait phenotypes, $\mu$ is the intercept, $\textbf{X}$ is an $n \times m$ matrix of marker genotype covariates, with *n* being the number of individuals, *m* the number of markers, and $\varvec{\alpha }$ is an $m \times 1$ vector of unknown random marker effects. In BayesC$\pi$, the prior for the $m \times 1$ marker effects is assumed to be identically and independently distributed as a mixture between a point mass at zero with probability $\pi$ and a normal distribution with null mean and variance $\sigma ^2_\alpha$, i.e.

12 $$\begin{aligned} \alpha _j \vert \pi , \sigma ^2_{\alpha } = {\left\{ \begin{array}{ll} 0 & \hbox { with probability}\ \pi \\ \sim N(0,\sigma ^2_{\alpha }) & \hbox { with probability}\ (1-\pi ) \end{array}\right. } \end{aligned}$$

The prior for $\sigma ^2_{\alpha }$ was a scaled inverse chi-square with $\nu _\alpha = 4.2$ degrees of freedom and scale parameter $S^2_{\alpha } =\frac{\sigma ^2_\alpha (\nu _\alpha -2)}{\nu _\alpha }$. The variance of marker effects, $\sigma ^2_{\alpha }$, is related to the additive genetic variance explained by the markers, $\sigma ^2_g$, as

$$\begin{aligned} \sigma ^2_g = \sigma ^2_\alpha (1-\pi )\sum _{j=1}^k2p_j(1-p_j), \end{aligned}$$

where $p_j$ is the allele frequency at marker *j*, which was assumed to be 0.5 for all markers. The probability $\pi$ was assumed to have a uniform(0,1) prior, the residual effects a $N(0,\sigma ^2_{e})$ prior, and the prior for $\sigma ^2_e$ was scaled inverse chi-square with $\nu _e = 4.2$ degrees of freedom and scale parameter $S^2_e =\frac{\sigma ^2_e(\nu _e -2)}{\nu _e}$.

The software package JWAS [21] was used to apply BayesC$\pi$ [5] to the 1,225 lines coloured green in Fig. 1. In BayesC$\pi$, MCMC samples are drawn for all the unknowns in the model, including the marker effects. To make inferences on $k^2_s$, markers with non-null effects were identified in each sample. The genetic variance in generation 0 was used as the initial value for the genetic as well as for the residual variance. The initial value for $\pi$ was taken as 1 minus the number of QTL divided by the total number of loci. MCMC sampling with a chain length of 510,000 samples was used, with the first 10,000 samples discarded as burn-in. The set of marker covariates with non-zero effects was stored for every $500^{th}$ sample.

### Estimating predictability in the simulated data

To examine the dependence between the number *n* of rows in the $n\times {s}$ trait-specific matrix $\textbf{X}_s$ of marker covariates and the number *s* of trait-specific markers on $k^2_s$, $n=10, 100, 1,000$ covariate vectors were randomly drawn without replacement from Syn-1-DH, coloured in green in Fig. 1. This was repeated for each of the three traits determined by 10, 100, or 1,000 QTL. The number of linearly independent rows in $\textbf{X}_s$ determines its row space. Thus, increasing the number *n* of rows in $\textbf{X}_s$ increases the probability that the vector of trait-specific marker covariates for a candidate is in the row space of $\textbf{X}_s$. From each of the candidate sets coloured in blue in Fig. 1, 250 marker covariate vectors were sampled without replacement to serve as candidates. Recall that for each of the three traits, 1,000 MCMC samples of marker covariates with non-null effects were available. For each of these samples, the mean of $k^2_s$ was calculated across all 250 candidates as $\bar{k^2_s} = \frac{1}{250}\sum _{j=1}^{250} k^2_{s_j}$, using the trait-specific $\textbf{X}_s$ and the sample-specific marker covariate vector with non-null effects for candidate *j*. These 1,000 $\bar{k^2_s}$ values were used to estimate the posterior distribution of $\bar{k^2_s}$ for each of the three traits and $n=10, 100, \text { or } 1,000$. These distributions were computed for each candidate sets in Fig. 1.

## Results and discussion

The objective of this paper was to show that the effective number $Q_e$ of QTL for a trait is inversely associated with the trait-specific genomic relationship between a candidate and the training set. This relationship explains how the number of QTL underlying a trait can affect the accuracy of prediction, for example, as observed in [16], when the candidates were not in the training set.

### Inverse association of $Q_e$ with accuracy of genomic prediction

The genomic relationship between the candidate and the training set has been recognized as an important factor that determines the accuracy of genomic prediction [9, 13]. In this paper we showed that the breeding value of a candidate can be decomposed into two components, $u_p$ and $u_r$, where $u_p$ is correlated with the phenotypes of the training individuals and $u_r$ is uncorrelated to these phenotypes. Further, we showed that the $k^2$ statistic proposed by [8] is equal to the squared correlation between *u* and $u_p$, which we defined as predictability. We also extended predictability to be trait specific.

#### Candidate is in the training set

From equations (2) and (5), we can see that when the candidate’s $\textbf{k}$ is in the row space of $\textbf{X}$, $\textbf{k}_p = \textbf{k}$ and $k^2 = \frac{\textbf{k}'_p\textbf{k}_p}{\textbf{k}'\textbf{k}}$ becomes 1.0. Recall that the results in Table 1 of [16] were for the situation where all candidates were from the training set and $k^2$ for all candidates would have been 1.0. Thus, in this case, the genomic relationship between the candidate and the training set, as quantified by $k^2$, will not depend on the number of QTL underlying the trait. This agrees with the results in Table 1 of [16], where the accuracy of prediction did not depend on the number of QTL.

#### Candidate is not in the training set

On the other hand, results presented in Table 2 of [16] were for candidates that were not in the training set, but where most of them were even from different breeds. Thus, most of the candidates were very distantly related to the training set. Recall that we used *s* to denote the number of trait-specific markers that capture the variability due to the QTL for the trait, where the number of such markers is associated with the effective number $Q_e$ of QTL. Regardless of the number of markers used for training and prediction, predictability depends only on the set of *s* trait-specific markers. Equivalently, even when $\textbf{X}$ is used for prediction, predictability depends only on $\textbf{X}_s$,  which has the set of *s* trait-specific markers. So, if $s \le n$, where *n* is the number of rows in $\textbf{X}_s$, it is possible that $\textbf{X}_s$ has row rank *s*. Given that $\textbf{X}_s$ has row rank *s*, any candidate vector $\textbf{k}_s$ of covariates will be in the row space of $\textbf{X}_s$ and thus, will have $k^2_s = 1.0$. However, if $s> n$, it is impossible for $\textbf{X}_s$ to have row rank *s*. In this case, the *s* elements that correspond to the trait-specific markers in the $\textbf{k}$ vector of a candidate may not be a linear combination of the $n\times s$ trait-specific marker covariate matrix, $\textbf{X}_s$. Even in this situation, the *s* elements that correspond to the trait-specific marker effects in the $\textbf{k}$ vector of a candidate may be in the row space of $\textbf{X}_s$, especially if the candidate is closely related to or included in the training set. Even when the *s* trait-specific elements of $\textbf{k}$ are not in the row space of $\textbf{X}_s$, $k^2_s$ is likely to be close to 1.0 for a candidate that is closely related to the training set, but the value of $k^2_s$ is expected to be lower for individuals that are distantly related to the training set. As *s* increases in size relative to *n*, it becomes less likely that the *s* trait-specific elements of $\textbf{k}$ of a candidate will be in the row space of $\textbf{X}_s$, and thus the expectation for the value of $k^2_s$ becomes smaller. As the results in Table 2 of [16] were mostly for distantly related candidates, we expect that $k^2_s$ would be inversely associated with *s*, which is directly associated with the effective number $Q_e$ of QTL. Thus, this inverse association of $k^2_s$ with $Q_e$ explains the result in [16] that accuracy of prediction was higher for traits with lower numbers of QTL.

### Disentangling the two contributions to the inverse association between the accuracy of prediction and $Q_e$

Another possible explanation for the inverse association between the number of QTL and accuracy of prediction is that effects of markers (or QTL) are better estimated when there are a few QTL with large effects than when there are many QTL with small effects. Below, we will compare the results in Tables 1 and 2 of [16] to disentangle the contributions from these two explanations for this inverse association.

The implicit assumption in the second explanation given above is that the QTL are known, and then, the signal to noise ratio is more favorable when few markers (or QTL) are fitted in the model compared to when many are fitted. In Table 1 of [16], where the candidates were in the training set, $k^2$ would be 1.0 for all candidates regardless of the number of QTL. So, in this table, differences in accuracy associated with the number of QTL are entirely due to the second explanation. First, consider the case where the QTL positions are known and QTL genotypes are fitted in the model. In the multibreed population considered by [16], the accuracy was 0.965 with 50 QTL and dropped to 0.810 with 500 QTL. Similarly, in the purebred population, the accuracy dropped from 0.978 to 0.877. In Table 2 of [16] the candidates were not in the training set and $k^2$ depends on the number of QTL. In this case, in Table 2, the corresponding accuracies considered above in Table 1 were all lower. This is because $k^2$ is not expected to be 1.0 between the candidates and the training set. Further, in Table 1 of [16], the average drop in accuracy was 0.13 compared to an average drop of 0.2 in Table 2. Here, the inverse association of the trait-specific genomic relationship of a candidate to the training set (as quantified by $k^2$) contributed to the drop in accuracy, in addition to the less favorable signal to noise ratio when the number of QTL increased from 50 to 500. Next, consider the case where the QTL are not known and all 50k makers are included in the model, regardless of the number of QTL. Here, regardless of the number of QTL underlying the trait, the signal to noise ratio is expected to be the same: 1) because the simulated signal (genetic variance) from 50 or 500 QTL was the same; and 2) because all 50k markers contribute to the noise in the predictions, regardless of the number of QTL. Thus, as expected, in Table 1 of [16], the average drop in accuracy when going from 50 to 500 QTL was close to zero, because neither of the two possible explanations for the inverse association between the number of QTL and accuracy of predictions are expected to come into play in this setting. In contrast, in Table 2 of [16], when all 50k markers were included in the model, the average drop in accuracy when going from 50 to 500 QTL was 0.15. Here, this entire drop can be attributed to the inverse association between the number of QTL and the trait-specific genomic relationship between the candidates and the training set, because the signal to noise ratio is expected to be the same for all analyses with the same number of markers in the model. In most practical situations, the QTL are not known, and in this case, the inverse association between the number of QTL and accuracy of prediction is entirely due to the inverse association between the number of QTL and the genomic relationship of candidates to the training set.

### Constructing training set to maximize $k^2$ or genetic diversity

Recall that the genomic relationship quantified by $k^2_s$ is not between two individuals but between a candidate and the entire training set. This is equally true for $k^2$, and thus, without loss of generality, we will consider how the genomic relationship quantified by $k^2$ can be changed by changing the training set. For example, the relationship to the training set of any candidate, even one from a distant breed that is not represented in the training set, can be increased by increasing the number of linearly independent rows in $\textbf{X}$, i.e., by increasing its rank. This can be done by sequentially adding one individual at a time to the training set. Suppose $n_c$ additional individuals can be phenotyped, and we need to choose those $n_c$ individuals from among $n_t$ individuals that have already been genotyped. This can be done as follows. Using the current value of $\textbf{X}$, $k^2$ is calculated for all $n_t$ individuals and the individual with the lowest value of $k^2$ is added to the training set. Then, $\textbf{X}$ is updated for the individual that was just added to the training set. This process is repeated until $n_c$ candidates have been added to the training set. This procedure can also be used to select from a population a subset of individuals with maximum genetic diversity.

### Simulation results on the assocations of $k^2_s$ with $Q_e$ and size of training set

In this paper, a simulation was used to demonstrate the dependence of $k^2_s$ on *s* relative to *n*, where *s* is the number of trait-specific markers, which is closely associated to $Q_e$ and the number of QTL. In contrast to the situation in [16], as shown in Fig. 1, the candidates in our maize-breeding simulation, colored in green, were closely related to the training set, colored in blue. Further, to reduce computing time for the simulation, only two maize chromosomes were simulated, the first with length 3.07 Morgans and the second with length 2.24 Morgans. Given that only one crossover is expected per Morgan, many of the related individuals among the 1,225 training individuals, which originated from 393 Petkuser lines (Fig. 1), are expected to share large independently segregating chromosomal segments. As a result, although we simulated up to 1,000 QTL, the number $Q_e$ was much lower ($<100$). This is due the high linkage disequilibrium (LD) that was present in the commercial maize line used to simulate the data. The effective number $Q_e$ of QTL has an upper bound equal to the number of independently segregating chromosomal segments, which is related to the level of LD. So, even when we attempted to simulate a 1,000 QTL, we ended up simulating less than 100 effective QTL. Thus, to examine the dependence of $k^2_s$ on *s*, very low values for *n* ($<100$) had to be considered.

**Fig. 2:**
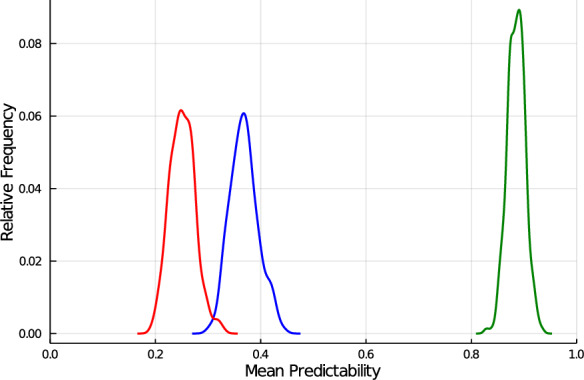
Distribution of the mean predictability for candidates in Syn-3 across 5 repetitions of the simulation, where 10 QTL (green graph), 100 QTL (blue graph) or 1,000 QTL (red graph) were underlying the trait. In all these situations, the training set size was 10. Predictability shows an inverse association with the number of QTL underlying a trait

Three traits, with 10, 100 or 1,000 QTL underlying the trait, were simulated, and training set sizes of 10, 100 or 1,000 observations were considered. The posterior distributions for the mean value of $k^2_s$ for the different candidate sets, colored in green in Fig. 1, were very similar due to their close genomic relationships to the training set. Further, because the effective number of QTL was less than 100, $k^2_s$ was 1.0 when $n\ge 100$. This shows the positive association between $k^2_s$ and *n*. Thus, results are presented in Fig. 2 for candidate set Syn-3, which is set that is most distantly related to the training set, for $n=10$ only. Results for all candidate sets are presented in Additional file 1: Fig. S1.

The distribution for $k^2_s$ colored in green in Fig. 2 is for the trait with 10 simulated QTL and $n=10$. The average of the number *s* of trait-specific markers from the 1,000 saved MCMC samples from BayesC$\pi$ was 12.9, which may indicate that more than one marker was needed to track some QTL. In samples where *s* was smaller than $n=10$, it is possible that $\textbf{X}_s$ had row rank equal to *s*. Whenever this is the case, $k^2_s$ will be 1.0 for all candidates. In samples where *s* was larger than $n=10$, $\textbf{X}_s$ cannot have row rank of *s*, but $k^2_s$ can still be 1.0, for example for a candidate that is in the training set. In this case, when $s>n$, however, it is possible for $k^2_s$ to take on values lower than 1.0. Here, where the mean value of *s* was only slightly larger than *n*, the mean value of $k^2_s$ was 0.9, which indicates that the vector of trait-specific marker covariates for a candidate was well represented as a linear combination of the rows of $\textbf{X}_s$ for most of the candidates considered. This implies that most candidates had a close trait-specific genomic relationship to the training set.

The distribution for $k^2_s$ colored in blue in Fig. 2 is for the trait with 100 simulated QTL and $n=10$. In this case, contrary to the situation with 10 QTL, the number *s* of trait-specific markers was on average smaller (51.2) than the number of simulated QTL. The minimum value for *s* from the 1,000 MCMC samples was 18. Thus, there were no samples with *s* smaller than $n = 10$, indicating that in none of the samples $\textbf{X}_s$ could have had a row rank of *s*. In this distribution, the mean value of $k^2_s$ was 0.37, indicating that the vector of trait-specific marker covariates for a candidate could not be represented well as a linear combination of the rows of $\textbf{X}_s$ for most of the candidates considered here. This implies that most candidates had a distant trait-specific genomic relationship to the training set with $n=10$.

The distribution for $k^2_s$ colored in red in Fig. 2 is for the trait with 1,000 simulated QTL and $n=10$. The number *s* of trait-specific markers was on average 91.8, which is much smaller than the number of simulated QTL, and the minimum number for *s* was 35. Thus, with $n=10$, as in the case with 100 QTL (blue distribution), in none of the samples could $\textbf{X}_s$ have had a row rank equal to *s*. In this distribution, the mean value of $k^2_s$ was 0.25, indicating that the vector of trait-specific marker covariates for a candidate was not well represented as a linear combination of the rows of $\textbf{X}_s$ for most of the candidates. As in the case with 100 QTL, this implies that most candidates had a distant trait-specific genomic relationship to the training set with $n=10$.

This simulation was able to demonstrate the inverse association between $Q_e$ and $k^2$, which quantifies the genomic relationship of a candidate with the individuals in the training set. To demonstrate this inverse association, however, we had to limit the training set size to an unrealistically low value of $n=10$. This was due the close relationship of the candidates with the training set. When the candidates are closely related to the individuals in training set, the accuracy of prediction is expected to be high, and thus, $k^2$, which is the upper bound of accuracy, is also expected to be high. It then follows that $Q_e$ is going to be low, as in our simulation, because of its inverse association with $k^2$. In contrast to our simulation, in Table 2 of [16] the candidates were distantly related to the training set, and the results presented in that table show the inverse association of $Q_e$ with $k^2$ for more realistic values of *n* of about 1,000. This demonstrates that the inverse association between $Q_e$ and the genomic relationship of a candidate with individuals in the training set, as quantified by $k^2$, is true in general and is not limited to low values of *n*.

## Conclusions

The main conclusion of this paper is that the trait-specific genomic relationship between a candidate and the training set, as quantified by $k^2_s$, is inversely associated with the effective number $Q_e$ of QTL. Thus, $k^2_s$ is also inversely associated indirectly with the actual number $N_{\text {QTL}}$ of QTL, although the inverse association of $k^2_s$ with $N_{\text {QTL}}$ is observed only when $N_{\text {QTL}} < M_e$. Further, there is a positive association of the trait-specific genomic relationship of a candidate with the size of the training set. In general, these associations of the number of QTL and the size of the training set with the trait-specific genomic relationship are only observed when 1) the candidate is not in the training set, and 2) the size of the training set is smaller than the number *s* of trait-specific markers, which is an upper bound for the effective number $Q_e$ of QTL.

## Supplementary Information

Additional file 1: Figure S1. Distribution of the mean predictability for candidates in Syn-1-DH, Syn-2, Syn-3, Syn-2-DH and Syn-2* across 5 repetitions of the simulation, where 10 QTL(first graph), 100 QTL (second graph) or 1,000 QTL (third graph) are underlyingthe simulated trait. Predictability reaches 1.0 when the number of observations usedfor training was ≥ 100, indicating, that in these situations, all candidate’s covariatevectors were in the row space of Xs. The inverse association of predictability withthe number of QTL is shown for n = 10 across the 3 graphs. Posterior distributionsfor the different candidate sets were largely overlapping indicating similar genomicrelationship to the training set.

## Data Availability

Simulated data available upon request
