# Supplementary figures and images for: On the inverse association between the number of QTL and the trait-specific genomic relationship of a candidate to the training set
Source: Genet Sel Evol. 2024 Dec 13;56:75. doi: 10.1186/s12711-024-00940-4 (PMC11639121; doi:10.1186/s12711-024-00940-4)

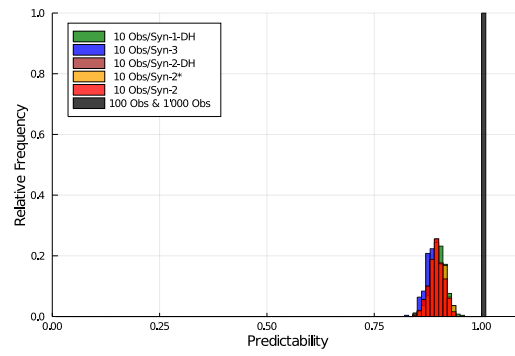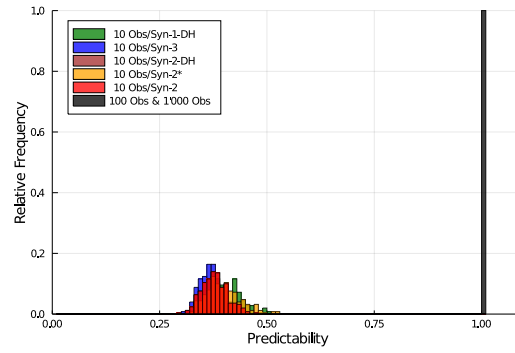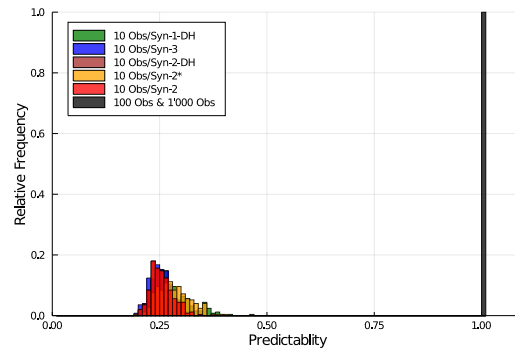

Supplement: Supplementary file 1 — Additional file 1: Figure S1. Distribution of the mean predictability for candidates in Syn-1-DH, Syn-2, Syn-3, Syn-2-DH and Syn-2* across 5 repetitions of the simulation, where 10 QTL(first graph), 100 QTL (second graph) or 1,000 QTL (third graph) are underlyingthe simulated trait. Predictability reaches 1.0 when the number of observations usedfor training was ≥ 100, indicating, that in these situations, all candidate’s covariatevectors were in the row space of Xs. The inverse association of predictability withthe number of QTL is shown for n = 10 across the 3 graphs. Posterior distributionsfor the different candidate sets were largely overlapping indicating similar genomicrelationship to the training set. [file 12711_2024_940_MOESM1_ESM.pdf]
